# Sweet Basil Functional Quality as Shaped by Genotype and Macronutrient Concentration Reciprocal Action

**DOI:** 10.3390/plants9121786

**Published:** 2020-12-16

**Authors:** Michele Ciriello, Antonio Pannico, Christophe El-Nakhel, Luigi Formisano, Francesco Cristofano, Luigi Giuseppe Duri, Fabiana Pizzolongo, Raffaele Romano, Stefania De Pascale, Giuseppe Colla, Mariateresa Cardarelli, Youssef Rouphael

**Affiliations:** 1 Department of Agricultural Sciences, University of Naples Federico II, 80055 Portici, Italy; michele.ciriello@unina.it (M.C.); antonio.pannico@unina.it (A.P.); christophe.elnakhel@unina.it (C.E.-N.); luigi.formisano3@unina.it (L.F.); francesco.cristofano@unina.it (F.C.); luigigiuseppe.duri@unina.it (L.G.D.); fabiana.pizzolongo@unina.it (F.P.); raffaele.romano@unina.it (R.R.); depascal@unina.it (S.D.P.); 2 Department of Agriculture and Forest Sciences, University of Tuscia, 01100 Viterbo, Italy; giucolla@unitus.it; 3 Consiglio per la Ricerca in Agricoltura e L’Analisi Dell’Economia Agraria, Centro di Ricerca Orticoltura e Florovivaismo, 84098 Pontecagnano Faiano, Italy

**Keywords:** *Ocimum basilicum* L., nutrient solution management, gas chromatography, volatile compounds, caffeic acid, chicoric acid, rosmarinic acid

## Abstract

Basil (*Ocimum basilicum* L.) is among the most widespread aromatic plants due to its versatility of use and its beneficial health properties. This aromatic plant thrives in hydroponics, which is a valid tool to improve the production and functional quality of crops, but nevertheless, it offers the possibility to de-seasonalize production. A floating raft system was adopted to test the production and quality potential during autumn season of three different genotypes of Genovese basil (Aroma 2, Eleonora and Italiano Classico) grown in three nutrient solutions with crescent electrical conductivity (EC: 1, 2 and 3 dS m^−1^). The aromatic and phenolic profiles were determined by GC/MS and HPLC analysis, respectively. The combination Aroma 2 and the EC 2 dS m^−1^ resulted in the highest production, both in terms of fresh weight and dry biomass. The 2 dS m^−1^ treatment determined the major phenolic content, 44%, compared to the other two EC. Italiano Classico showed a higher total polyphenolic content in addition to a different aromatic profile compared to the other cultivars, characterized by a higher percentage of Eucalyptol (+37%) and Eugenol (+107%) and a lower percentage of linalool (−44%). Correct management of the nutritional solution combined with adequate genetic material managed an improvement in the production and the obtainment of the desired aromatic and phenolic profiles.

## 1. Introduction

Basil (*Ocimum basilicum* L.) is undoubtedly a transcendent aromatic plant of the Lamiaceae family [1]. Despite its tropical origin, basil is widely used in Mediterranean cuisine as an indispensable ingredient of traditional dishes due to its fresh and aromatic leaves [2]. Apart from its gastronomic value, the pharmaceutical and cosmetic industries have made basil a “versatile herb”, sought for its distinctive chemical composition, which includes volatile compounds and phytochemicals beneficial for human health [3,4,5]. Several studies highlighted that bioactive compounds such as rosmarinic acid, caffeic acid and chicoric acid [4,6,7,8,9] confer to basil treasured antioxidant, antiviral, antibacterial, antimutagenic and anti-allergic properties [10,11,12,13]. The affluent and intense aromatic profile of basil leaves represents a distinctive quality attribute over other aromatic herbs [14]. This aromatic profile is mainly characterized by the presence of phenylpropanoids (e.g., estragole, eugenol and methyl-eugenol) and monoterpenes (e.g., linalool and eucalyptol) produced by the leaves and secreted through dedicated structures known as peeled glandular trichomes [15].

Chadha and Rajendra [16] identified 160 species of *Ocimum*, characterized by a great genetic variability that influences the morphological characteristics, composition and concentration of odorous molecules and the accumulation of secondary metabolites such as phenols [17,18]. However, in addition to the genetic aspect, the accumulation and biosynthesis of these biomolecules is influenced by physiological and environmental factors such as climate, cultivation technique, plant nutrition, phenological phase, environmental stress, plant ontogenesis and their mutual interactions [3,17,18,19]. For example, studies conducted by Di Cesare et al. [20,21] showed an evident influence of the environment on the aromatic profile of Genovese basil. For instance, basil plants cultivated in Liguria (Italy) showed a prevalence of linalool, eucalyptol, eugenol and methyl-eugenol that is not present in basil leaves grown in other regions of Italy. A further study conducted by Hussain et al. [22] showed that the chemical composition of essential oils varies according to the harvest season, with a prevalence of sesquiterpenes in summer and oxygenated monoterpenes in winter.

Basil is a versatile leafy vegetable for which demand is constantly increasing [23]. Notwithstanding, ensuring its off-season production with high quality is not simple with traditional cultivation methods. Consumers’ interest in fresh leafy vegetables united with their eco-sustainable and high-quality features has prompted producers to develop innovative production systems. Therefore, the floating raft system is a valid alternative for short cycle cultivation that occurs for basil. Control of the growing parameters through careful management of the nutrients, absence of soil and telluric organisms and recycling of the nutrient solution ensure high yields and high-quality attributes due to the accumulation of secondary metabolites with antioxidant activity and flavour enhancers [3,24]. Indeed, among the preharvest factors, mineral nutrition management is one of the most effective methods to improve the yield and functional quality of horticultural products [25]. In this regard, hydroponics represents a key tool, as it provides accurate control of the nutritional status of the plant [26,27]. Moreover, the shortage of agricultural land makes soilless cultures an interesting alternative to traditional open field cultivation, as it is well suited to constantly developing urban areas [28]. Additionally, Saha et al. [28] observed that soilless cultivation of basil guarantees a higher marketable yield compared to the open field. Furthermore, Sgherri et al. [10] detected a higher antioxidant activity of sweet basil grown hydroponically than those grown in soil. In soilless cultures, nutrient solution management is a critical preharvest factor that plays an imperative role in plant growth. The concentration and composition of the nutrient solution can modulate the organoleptic, functional and productive attributes of vegetable. Herein, positive nutritional stress can lead to increased vegetable quality due to physiological adjustments and accumulation of secondary metabolites as an adaptive response to suboptimal nutritional levels [27].

The reviewed scientific literature showed a positive correlation between modulation of the nutrient solution and biosynthesis of secondary metabolites which provide a qualitative boost [3,27,29,30,31]. Nevertheless, the lack of specific information about the optimal macronutrient concentration of the nutrient solution as well as the outcomes on production and quality attributes of sweet basil requires further investigation. Our research aimed to assess the impact of three nutrient solutions with different macronutrient concentrations (1 dS m^−1^, 2 dS m^−1^ and 3 dS m^−1^) on the production, aromatic and phenolic profiles of three different Genovese basil cultivars grown in a floating raft system, thus identifying the best combination of nutrient solution and Genovese basil cultivar to guarantee in the autumn season a correct balance between production and quality and, concomitantly, ensuring a low environmental impact and paving the way for future work.

## 2. Results

### 2.1. Biometric Measurements, Dry Biomass and Dry Matter Percentage

As illustrated in Table 1, only the mean effect of the cultivars determined the difference in leaf number per plant, where Aroma 2 registered the highest value followed by Eleonora and then Italiano Classico. However, the interaction between the two examined factors (cultivar and nutrient solution) was dominant for the rest of the morphometric parameters. The combination Aroma 2 × NS 2 dS m^−1^ guaranteed the highest production of fresh and dry biomass (3.36 and 0.25 kg m^−2^, respectively). The same cultivar grown in nutrient solution at 1 dS m^−1^ presented the highest dry matter value (7.97%), while the lowest percentage value was displayed by Italiano Classico grown in nutrient solution, 1 dS m^−1^ (5.91%), whereas Elenonora maintained a steady dry matter between all of the different nutrient solutions. Eleonora and Italiano Classico, both cultivated in medium concentration NS (2 dS m^−1^), recorded the highest number of nodes per plant and the highest leaf/stem ratio, respectively. Regardless of the used nutrient solution, Aroma 2 showed the lowest values for the latter parameter.

### 2.2. Colorimetric Indices, SPAD Index and Fluorescence

According to Table 2, the statistical analysis listed significant variations among basil cultivars for all colorimetric components. As far as the L* (brightness) parameter is concerned, only the cultivar mean effect was significant, the highest values were obtained in both Eleonora and Italiano Classico. The latter cultivar had the highest b* value and an absolute negative value for a*. The macronutrients concentration in the nutrient solution significantly influenced the parameters a * and b*. The increase in electrical conductivity of the nutrient solution resulted in a 17% increase in a* values. On the other hand, the switch from the most concentrated to the most diluted nutrient solution produced a 29% increase for b* values, meaning an increase towards the yellow axe, which is inversely correlated with the SPAD index that decreased with reduction of the macroelements. The Fv/Fm ratio, which defines the fluorescence index, was significantly influenced by both the cultivar and the nutrient solution but not by their interaction. Particularly, Italiano Classico and a concentration of 1 dS m^−1^ defined the lowest values. Concerning the SPAD index, the interaction of the two considered factors was significant: the combinations Aroma 2 × NS 2 and 3 dS m^−1^, and Eleonora × NS 3 dS m^−1^ showed the highest SPAD values (avg. 33.0).

### 2.3. Sweet Basil Aromatic Profile

The solid phase microextraction (SPME)-GC/MS analysis of volatile components enabled determination of the aromatic profile of basil cultivars subjected to three different concentrations of macronutrients. In Table 3, the percentage of the seven main compounds is reported. Among these, eucalyptol, linalool and eugenol constituted, on average, about 70% of the whole aromatic profile, followed by 1-Octen-3-ol, α-bergamotene, trans-2-hexenal and β-cis-ocimene. The genotype significantly influenced biosynthesis of all reported odorous molecules. Italiano Classico was characterized by a higher amount of eucalyptol (+37%) and eugenol (+107%) but by a lower percentage of linalool (−44%), compared to the other two cultivars. On the other hand, the interaction between the two considered factors determined relevant differences between the following compounds: trans-2-hexenal, eugenol and α-bergamotene. In general, Italiano Classico reached the highest levels of trans-2-hexenal and eugenol. Specifically, the transition from the concentrated nutrient solution to the diluted one determined an increase of about 118% for eugenol. Instead, trans-2-hexenal showed an opposite trend, with the highest percentage obtained from the combination Italiano Classico × 3 dS m^−1^. Use of the most concentrated solution determined the highest percentage of α-bergamotene for Eleonora, whereas this same NS gave similar percentages to the 2 dS m^−1^ solution for the other two cultivars.

### 2.4. Sweet Basil Phenolic Acids

The phenolic profile of the three Genovese basil cultivars grown with different concentrations of macronutrients (Table 4) revealed significant differences. The main abundant phenolic acids in basil were rosmarinic acid followed by chicoric acid and caffeic acid, with Eleonora accumulating the least polyphenols in general and Italiano Classico accumulating the most. A significant interaction between the cultivars and the nutrient solutions was noted for all phenolic acids. Eleonora showed no significant improvement in its total polyphenols content in any of the NS treatments. Moreover, the highest accumulation of caffeic acid and chicoric acid was noted in the combination Italiano Classico × 1 dS m^−1^, whereas a combination of the same cultivar with 2 dS m^−1^ NS resulted in the highest value of rosmarinic acid (512.2 µg g^−1^ dw). However, the higher content of the less represented ferulic acid was obtained from the combination Aroma 2 × 2 dS m^−1^. Overall, the accumulation of the polyphenols was triggered for only Aroma 2 and Italiano Classico, with different combinations, where the best combination for a higher content was revealed with Italiano Classico × 2 dS m^−1^ (804.1 µg g^−1^ dw).

## 3. Discussion

The best production and quality performance combined with the efficient use of water and nutrients that characterize closed-loop soilless cultivation [32] have captured the attention of the entire agricultural sector. Among the various pre-harvesting factors, the genetic starting material and the nutrition management portray crucial factors in defining quality parameters [25]. Whilst the choice of genotype is dictated by the final destination of the product, hydroponics represents a valid tool, as it guarantees precise control of plant nutrition, thus offering many advantages over traditional cultivation in agricultural soil [24]. In the present experiment, the sweet basil cultivars grown in a floating system determined on average a yield about 7 times higher than the production of 38 basil cultivars grown in soil [28]. This result is mainly justified by the adopted high density (317 m^−2^), which features cultivation of basil on floating panels [33,34]. Although numerous studies underlined how the composition and concentration of the nutrient solution affects growth and yield [35,36,37], in this case, the average nutrient solution effect did not lead to differences in fresh production, leaf number and dry matter percentage. It can be partly attributed to the moderate salinity tolerance of basil [38,39] but, above all, to the short growing cycle (34 DAT), which minimizes the influence of macronutrients concentrations in the solution on growth parameters [26]. These results are also confirmed by the Fv to Fm ratio that showed a similar efficiency of the PSII system for all the applied treatments [40] without any evidence of stress or deficiency. This confirms the light significant differences in yield and growth parameters between the treatments. On the contrary, the cultivar mean effect clearly influenced all the measured morphometric parameters, underlining again the key role played by genetics. In particular, Aroma 2 was characterized by a higher percentage of dry matter and a lower leaf/stem ratio (in weight) resulting from a higher production of leaves per plant regardless of the nutrient solution. The latter two aspects represent technological characteristics greatly sought after by the agro-industry, as the excessive presence of the stem could increase the processing time due to its greater fibrousness, thus determining an early blackening of “pesto” [2]. Identically to the observation of Maggio et al. [41], an interaction between the genetic background of the different cultivars and the different concentrations of the nutrient solution was obvious, specifically in the case of fresh and dry biomass. Each cultivar achieved the best production with a specific nutrient solution, highlighting how tolerance to the concentration of salts in the nutrient solution is highly dependent on the genotype [38,42]. The use of nutrient solution 2 dS m^−1^ determined on average an increase of Aroma 2 fresh biomass of about 30% compared to those recorded with the other two nutrient solutions, confirming how the yield is negatively affected by nutrient solutions that are too concentrated and/or too diluted [43]. On the contrary, the Eleonora and Italiano Classico cultivars reached the highest production of fresh and dry biomass when grown under sub- and supraoptimal nutrient conditions, at 1 and 3 dS m^−1^, respectively.

On the other hand, colour is an important quality characteristic for leafy vegetables as it influences consumers’ acceptability and preferences [44,45]. The evaluation of colorimetric parameters as well as the SPAD index revealed a strong influence by the cultivar. The leaves of Aroma 2 were characterized by a lower brightness (L*) and intensity of yellow (b*) but higher SPAD values. Instead, Italiano Classico had a higher intensity of green (a*) in absolute. The same parameters were also influenced by the concentration of macronutrients in nutrient solution as also observed by Maggio et al. [41] and by Walters and Currey [37]. It is noteworthy that the increase of the nutrient solution concentration, independent from the cultivar effect, determined a simultaneous increase of SPAD values and a decrease of the yellow intensity (b*). These results confirm what was observed by Fallovo et al. [43], where low concentrations of nutrients reduce the content of chlorophyll with subsequent yellowing (b*) due to lower levels of N that, in this case, characterized the most diluted nutrient solution (1 dS m^−1^). The interaction between the two considered factors defined significant differences only for SPAD values. SPAD is a nondestructive tool widely used to indirectly monitor the chlorophyll content of leaves [46,47]. The higher SPAD values obtained from the combination Aroma 2 × 2 dS m^−1^ could explain the higher production resulting from this combination.

On the other hand, a relevant quality aspect in an aromatic plant is certainly the composition of its aromatic profile, which indiscriminately defines its taste. The flavour of basil is mainly owed to the presence of odorous molecules produced and stored in peeled glandular trichomes located in the aerial parts of the plant [15]. The aromatic compounds characteristic of sweet basil belong to two distinct groups: terpenes and phenylpropenes that are synthesized by two different metabolic pathways [48]. As observed by several authors [15,18,49], the cultivars Eleonora and Aroma 2, used for the industrial production of “Pesto Genovese”, were characterized by a higher presence of linalool, oxygenated monoterpene that contributes to the good quality of the essential oil and therefore of the famous Italian sauce [5]. Nevertheless, Italiano Classico was characterized by a dominant presence of eucalyptol, a metabolite extremely interesting for its biological activity thanks to its wide use in the pharmaceutical field [18,50]. However, it was never indicated as a predominant aromatic compound of the Genovese type. However, considering that the two molecules linalool and eucalyptol have the same precursor (geranyl pyrophosphate (GPP)), the conversion to linalool and eucalyptol is mediated by specific enzymes (linalool synthase (LIS) and 1,8-cineol synthase), as suggested by Chang et al. [51], but could also be influenced in a genotype × environment interaction, since the different enzymatic activities are highly sensitive to environmental conditions [52]. In addition to the genetic and environmental component, plant nutrition is to be considered a further liable factor to quanti-qualitatively modify the aromatic profile [53]. The different concentrations of macronutrients in nutrient solution significantly influenced the content of trans-2-hexenal, α-bergamotene and eugenol. The latter aromatic compound is associated with sensory quality [5] and has a recognized antioxidant power [54]. As observed by Nurzyńska-Wierdak and Borowski [55], the content of this relevant aromatic molecule especially in Italiano Classico was negatively affected by the increased presence of the macrocations, whereas for Eleonora, the use of more concentrated nutrient solutions determined a linear increase of α-bergamotene. The greater biosynthesis of this sesquiterpene typical of sweet basil [56] could be related to the greater availability of potassium, which as always observed by Nurzyńska-Wierdak and Borowski [55], and Singh et al. [57] determined in *Ocimum basilicum* L. a greater production of aromatic molecules belonging to this chemical class.

The growing interest in this aromatic plant beyond the culinary field is mainly attributed to the high presence of a multitude of phenolic compounds considered as powerful antioxidants [9,58,59,60]. Phenolic compounds are secondary metabolites produced by plants through the shikimate pathway [61] and facilitate adaptation of the plant to the surrounding environment [57]. The content of phenolic composition, as shown by our results, is strongly influenced by the genetic aspect [58,62,63]. Regardless of the nutrient solution effect, Italiano Classico compared to Aroma 2 and Eleonora was characterized by higher total polyphenol concentrations of 147% and 783%, respectively. The phenolic profile of Italiano and Aroma 2 is in line with that reported in the literature [3,59,64], where a high content of rosmarinic acid is registered. This latter acid is an ester of caffeic acid, synthesized from the amino acids L-tyrosine and L-phenylalanine [61,65]. Rosmarinic acid is a characteristic secondary metabolite of sweet basil to which is attributed a high antioxidant capacity [66]. In general, the average concentration of rosmarinic acid of the two basil cultivars (Aroma 2 and Italiano Classico) grown in floating was lower than that obtained by Kiferle et al. [23] and Javanmardi et al. [59] but higher than that recorded by Sgherri et al. [10]. These results, besides underlining the influence of the genotype, may have been due to the different extraction methods and solvents used for the determination of such a phenolic acid [66] but, most importantly, to the different growth conditions. In this specific case, the lower production of phenolic compounds compared to basil grown in the open field [59] could be related to the different methods of cultivation. In fact, the hydroponic cultivation optimizing the growing conditions reduces the possibility of oxidative stress [10] and, therefore, the biosynthesis and the accumulation of phenolic compounds that are actively involved in neutralization of the free radicals formed just under conditions of oxidative stress [30]. Although rosmarinic acid is constantly defined as the most represented phenolic acid in basil [3,4,59,66], the results showed that the phenolic profile of Eleonora was characterized by a dominant presence of chicoric acid. The influence of the genotype on the major biosynthesis of this phenolic acid is confirmed by the work of Kwee et al. [67], where nine varieties of basil were identified with an absolute higher content of chicoric acid. The use of nutrient solutions with different concentrations of macronutrients determined significant differences for all the evaluated phenolic compounds, as nutritional eustress induces physiological responses and molecular mechanisms that cause accumulation or decrease of bioactive compounds needed by the plant to adapt to suboptimal conditions [68]. In line with previous work on both basil [3,69,70] and other vegetables of interest [25,68], the nutrient limitation, regardless of the cultivar effect, led to higher production of caffeic acid and chicoric acid. These results are not surprising since adverse conditions such as low nutrient levels, particularly N, intensify the activity of phenylalanine ammonia-lyase (PAL) and other enzymes regulating the biosynthesis of phenolic compounds [71,72]. In addition, these suboptimal nutrient conditions limit growth without blocking photosynthetic activity, resulting in excessive production of carbohydrates that could be partly converted into C-based secondary metabolites such as phenols [3]. Nevertheless, it should be noted that the use of the more diluted solution (1 dS m^−1^) did not increase the production of rosmarinic acid and ferulic acid, since as observed by Heimler et al. [73], the increase of secondary metabolites in conditions of nutritional deficit does not involve in general all phenolic compounds. Additionally, as observed by Jakovljević et al. [69], the decrease in the concentration of macroelements in the nutrient solution has not resulted in a greater accumulation of phenolic compounds for all cultivars, confirming how the availability of nutrients modulates the phenolic content in a genotype-dependent way [68,73]. That said, it is interesting to note that, for Italiano classic, the use of 2 dS m^−1^ NS has simultaneously reduced biomass production but increased the total polyphenols content, particularly affecting the concentration of rosmarinic acid. This result partially confirms how the accumulation of secondary metabolites could lead to a slower growth rate [61,74].

## 4. Materials and Methods

### 4.1. Basil Cultivars, Nutrient Solution Concentrations, Growing Conditions and Experimental Design

This research aimed to evaluate the end results of three nutrient solutions with different macronutrient concentrations on the quantitative and qualitative attributes of three Genovese basil cultivars in a floating raft system. The experiment was carried out in an unheated greenhouse located at the University of Naples Federico II, Department of Agriculture (DIA) in Portici (Naples, Italy; 40°48′ N, 14°20′ E, 29 m.s.l. Three cultivars of basil (*Ocimum basilicum* L.), Eleonora (Enza Zaden, Enkhuizen, Noord-Holland, The Netherlands), Aroma 2 (Fenix, Belpasso, Catania, Italy) and Italiano Classico (La Semiorto, Sarno, Salerno, Italy), were transplanted on 1 November 2019 in 54-hole polystyrene trays (52 × 33 cm) at a density of 317 plants m^−2^. Each experimental unit consisted of a tray containing 54 plants.

The experimental project was organized in a factorial design with three replicates, consisting of three cultivars (C) (i.e., Eleonora, Aroma 2 and Italiano Classico) and three nutrient solution (NS) strengths (i.e., 1 dS m^−1^, 2 dS m^−1^ and 3 dS m^−1^). The cultivation system consisted of twenty-four water tanks with a maximum capacity of 35 L, each filled with 30 L of NS. The macronutrient concentrations of the standard NS (i.e., 2 dS m^−1^) were 14.0 mM nitrate, 1.5 mM phosphorous, 3.0 mM potassium, 1.75 mM sulfur, 4.5 mM calcium, 1.5 mM magnesium and 1.0 mM ammonium, while the micronutrient concentrations were 15 μM iron, 9 μM manganese, 0.3 μM copper, 1.6 μM zinc, 20 μM boron and 0.3 μM molybdenum. The 1 dS m^−1^ and 3 dS m^−1^ NS concentrations were obtained by halving or increasing (×1.5) the macronutrients concentrations, respectively. During the experiment, the pH of the different NS was monitored every other day and kept around 5.8 ± 0.2 using a portable pH-meter (HI 991301, Hanna Instruments, Milan, Italy). To avoid anoxia in the aqueous medium, an immersion pump was used in each water tank. During the growing period, the greenhouse mean day/night air temperature was 23/13 °C.

### 4.2. Harvesting, Biometric Analysis and Sampling

Basil plants were harvested at 34 DAT, where part of the harvest was used to determine the main biometric indices: total fresh weight per square meter, leaf and node number per plant, leaf/stem ratio (by weight), percentage of dry matter, and total dry weight per square meter. The latter parameter was obtained by placing the plant material in a ventilated stove at a temperature of 70 °C for a total of 72 h. The remaining harvest was immediately placed in a freezer at −80 °C to preserve the quality of the final product.

### 4.3. Colorimetric Measurement, SPAD Index and Maximum Quantum Efficiency Determination

Prior to harvest, the colorimetric indices (L*, a* and b*) were evaluated with the Minolta Chroma meter CM-2600d (Minolta Camera Co. Ltd., Osaka, Japan). The instrument, composed of a portable spectrophotometer, was properly calibrated with the Minolta standard; then, the measurements were made on the top leaf blade of 15 fully expanded leaves per experimental unit. The measurements of the green index (SPAD) were made with a handheld Minolta Chlorophyll Meter SPAD-502 (Minolta Camera Co. Ltd., Osaka, Japan), taking the third fully expanded leaf from the top as reference. The observations involved 10 plants per experimental unit. Leaves from the same phenological stage were used for fluorescence measurements through a portable fluorometer (Plant Stress Kit, Opti-Sciences, Hudson, NH, USA). For each experimental unit, 4 measurements were made in the 11:00–13:00 timeslot. The maximum quantum efficiency of PSII photochemistry expressed as Fv/Fm was calculated as (Fm-Fo)/Fm, where Fo and Fm represented, respectively, the initial fluorescence and the maximum fluorescence of a sample adapted to darkness for 10 min. The fluorometer was calibrated using the “autoset” option on a leaf similar to the leaves to be measured. The instrument uses an algorithm that allows automatic optimal setting to the modulated light intensity and gain.

### 4.4. Extraction and Determination of Basil Aromatic Profile

Determination of the aromatic profile of basil was carried out by gas chromatography combined with the mass spectrometer (GC/MC) (Agilent, Santa Clara, CA, USA) after having extracted and concentrated the volatile molecules (VOC’s) using the solid phase microextraction (SPME) technique. A GC 6890N equipped with a 5973 mass spectrometer was used, while the spectrometer was set to 70 eV. The 10 mL vial containing 500 mg of fresh frozen basil was stirred continuously with a magnetic stir bar for 10 min (min) at 30 °C. Subsequently, 50/30 µm thick DVB/CAR/PDMS fibre (Supelco^®^,Bellofonte, PA, USA), coated with stationary phase, was introduced into the vial. After 10 min of contact, the SPME fibre was injected into the GC/MS injector where it underwent a thermal desorption of the analytes for 10 min at 250 °C. Split-less injection was used for the samples. The volatiles molecules were separated in a DB-5 capillary column (5% Phenyl, 95% dimethylpolysiloxane, 30 m × 0.250 mm, 0.25 µm; Agilent, Santa Clara, CA, USA). For the first 2 min, the furnace temperature was maintained at 50 °C and then increased to 10 °C min^−1^ from 50 °C to 150 °C and later from 150 °C to 280 °C to 15 °C min^−1^. The ion and injection source temperatures were 230 °C and 250 °C, respectively. Helium was used as a carrier gas at a flow rate of 1 mL min^−1^. The compounds were identified after verification with retention indexes using NIST Atomic Spectra Database version 1.6 (U.S. Department of Commerce, Gaithersburg, MD, USA). Three replicates were performed for each sample with results expressed in percentage (%).

### 4.5. Extraction, Determination and Quantification of Phenolic Acids

All reagents, standards and solvents were purchased from Sigma Aldrich (Milan, Italy) and were HPLC grade. Preparation of the phenolic extracts followed the method of Ferracane et al. [75] with minor modifications. 2 mL of 70% (*v*/*v*) methanol–water mixture was added to 100 mg of freeze-dried basil sample. This mixture was vortexed, sonicated and agitated for 1, 20 and 10 min, respectively. It was then centrifuged for 10 min at 6800 rpm and finally filtered with a 45-µm membrane filter (Phenomenex, Torrance, CA, USA). The supernatant was pipetted into a sterile falcon from amber glass and analysed to quantify the following compounds: caffeic acid, rosmarinic acid, chicoric acid and ferulic acid. The chromatographic separation of phenolic compounds using a 20-μL sample injection loop was performed using an Agilent 1100 Series HPLC system (Santa Clara, CA, USA) equipped with a degasser (G4225A), quaternary pump (G13111A) and diode matrix detector (G1315B). The used column was a reverse phase C18 (150 × 4.6 mm d.i.; particle size 5 μm; Kinetex^®^ 100 Å column C18; Phenomenex, Torrance, CA, USA), while the eluents were 0.1% (*v*/*v*) trichloroacetic acid in H_2_O (A) and acetonitrile (B). The gradient program set was 0–50% for 50 min at a constant flow of 1 mL/min. Detection of individual phenolic compounds was performed at 280 nm. Identification was then performed by comparing the retention times with the standards. Calibration curves were built for each standard using seven concentration levels (0.15, 0.5, 1, 10, 20, 50 and 100 mg·L^−1^). For each sample, three replicates were performed. Results were expressed as μg g^−1^ dry matter.

### 4.6. Statistical Analysis

All data were subjected to variance analysis (ANOVA) using the IBM SPSS 20 package (www.ibm.com/software/analytics/spss). For each significant identified variable (*p* < 0.05), a multiple Duncan interval test (DMRT) was performed.

## 5. Conclusions

Over the last few years, agriculture has been driven by the need to produce food for the growing global population, which has led to modules and cultivation techniques capable of increasing crop productivity without compromising sustainability and quality aspects. Due to the short cultivation cycle of sweet basil, the floating raft system would guarantee not only optimal yield but also the possibility to maximize production of secondary metabolites highly demanded by consumers and the pharmaceutical and cosmetic industries. The results obtained in this paper highlighted the productive potential of this alternative cultivation system for Genovese basil specially in the autumn season, where the obtained fresh yield (≈3 kg m^−2^) was higher than what is normally obtainable in the open field. Eleonora cultivar unexpectedly guaranteed high fresh biomass with the lowest EC, thus illustrating the possibility of reducing fertilizers use coupled with less demanding cultivars as an agronomic tool to lower the environmental impact. Italiano Classico revealed the highest total polyphenols content (804.1 µg g^−1^ dw) at 2 dS m^−1^ NS, while caffeic acid and chicoric acid production was maximized when grown at 1 dS m^−1^ NS; the same cultivar was characterized by a higher percentage of eucalyptol (+37%) and eugenol (+107%). Hence, the choice of genotype as well as the concentration of macronutrients depends on the target goal.

## Figures and Tables

**Table 1:** Sweet basil biometric parameters in light of the cultivar and the nutrient solution electrical conductivity.

Source of Variance	Leaf Number	Node Number	Fresh Yield	Dry Biomass	Leave/Stem Ratio	Dry Matter
	(No. Plant^−1^)	(No. Plant^−1^)	(kg m^−2^)	(kg m^−2^)		(%)
Cultivar (C)						
Eleonora	13.07 ± 0.32 b	4.83 ± 0.08 a	3.06 ± 0.05 a	0.20 ± 0.00 b	2.50 ± 0.05 b	6.44 ± 0.08 b
Aroma 2	16.94 ± 0.88 a	4.22 ± 0.05 b	2.87 ± 0.14 b	0.22 ± 0.01 a	1.94 ± 0.07 c	7.59 ± 0.13 a
Italiano Classico	10.65 ± 0.25 c	4.01 ± 0.01 c	3.04 ± 0.06 a	0.20 ± 0.01 b	2.72 ± 0.10 a	6.46 ± 0.14 b
Nutrient solution concentration (NS)						
1 dS m^−1^	12.99 ± 0.80	4.25 ± 0.10 b	2.93 ± 0.11	0.19 ± 0.00 b	2.30 ± 0.12	6.71 ± 0.33
2 dS m^−1^	14.08 ± 1.35	4.40 ± 0.17 a	3.06 ± 0.08	0.21 ± 0.01 a	2.44 ± 0.17	6.87 ± 0.12
3 dS m^−1^	13.60 ± 0.96	4.41 ± 0.12 a	2.98 ± 0.08	0.21 ± 0.00 a	2.42 ± 0.12	6.90 ± 0.15
C × NS						
Eleonora × 1 dS m^−1^	12.67 ± 0.61	4.63 ± 0.00 c	3.23 ± 0.03 ab	0.20 ± 0.01 c	2.63 ± 0.11 bc	6.23 ± 0.13 de
Eleonora × 2 dS m^−1^	13.42 ± 0.40	5.04 ± 0.11 a	2.97 ± 0.08 c	0.20 ± 0.00 cd	2.45 ± 0.10 c	6.57 ± 0.10 cd
Eleonora × 3 dS m^−1^	13.13 ± 0.75	4.83 ± 0.11 b	2.99 ± 0.02 c	0.19 ± 0.00 cd	2.42 ± 0.04 c	6.51 ± 0.08 cd
Aroma 2 × 1 dS m^−1^	15.58 ± 1.06	4.12 ± 0.01 e	2.54 ± 0.12 e	0.20 ± 0.01 c	1.88 ± 0.03 d	7.97 ± 0.23 a
Aroma 2 × 2 dS m^−1^	18.42 ± 2.21	4.17 ± 0.04 e	3.36 ± 0.09 a	0.25 ± 0.01 a	1.86 ± 0.05 d	7.32 ± 0.10 b
Aroma 2 × 3 dS m^−1^	16.83 ± 1.18	4.36 ± 0.08 d	2.72 ± 0.11 de	0.20 ± 0.01 c	2.08 ± 0.21 d	7.47 ± 0.12 b
Italiano Classico × 1 dS m^−1^	10.71 ± 0.48	4.01 ± 0.01 e	3.02 ± 0.01 bc	0.18 ± 0.00 d	2.39 ± 0.11 c	5.91 ± 0.06 e
Italiano Classico × 2 dS m^−1^	10.42 ± 0.67	4.00 ± 0.00 e	2.87 ± 0.04 cd	0.19 ± 0.00 cd	3.00 ± 0.07 a	6.73 ± 0.09 c
Italiano Classico × 3 dS m^−1^	10.83 ± 0.15	4.03 ± 0.03 e	3.24 ± 0.07 ab	0.22 ± 0.01 b	2.78 ± 0.02 ab	6.73 ± 0.04 c
Significance						
Cultivar (C)	***	***	*	***	***	***
Nutrient solution (NS)	ns	*	ns	**	ns	ns
C × NS	ns	*	***	***	**	***

ns, *, **, *** Nonsignificant or significant at *p* ≤ 0.05, 0.01 and 0.001, respectively. Different letters within each column indicate significant differences according to Duncan’s multiple-range test (*p* = 0.05). All data are expressed as mean ± se (n = 3).

**Table 2:** Colorimetric indices, SPAD index and fluorescence in light of the cultivar and the nutrient solution electrical conductivity.

Source of Variance	L*	a*	b*	SPAD Index	Fluorescence Fv/Fm
Cultivar (C)					
Eleonora	46.46 ± 0.22 a	−9.81 ± 0.31 b	25.98 ± 0.99 b	30.81 ± 0.46 b	0.81 ± 0.00 a
Aroma 2	44.69 ± 0.18 b	−6.76 ± 0.41 a	14.94 ± 1.28 c	32.54 ± 0.49 a	0.82 ± 0.00 a
Italiano Classico	46.12 ± 0.46 a	−11.27 ± 0.18 c	29.90 ± 0.87 a	30.63 ± 0.41 b	0.80 ± 0.00 b
Nutrient solution concentration (NS)					
1 dS m^−1^	45.67 ± 0.46	−10.06 ± 0.53 c	26.66 ± 1.93 a	30.08 ± 0.35 c	0.80 ± 0.00 b
2 dS m^−1^	45.83 ± 0.44	−9.21 ± 0.79 b	23.51 ± 2.63 b	31.54 ± 0.46 b	0.81 ± 0.00 a
3 dS m^−1^	45.77 ± 0.33	−8.57 ± 0.76 a	20.66 ± 2.35 c	32.37 ± 0.46 a	0.82 ± 0.00 a
C × NS					
Eleonora × 1 dS m^−1^	46.20 ± 0.64	−10.36 ± 0.63	28.53 ± 1.13	29.65 ± 0.47 c	0.81 ± 0.01
Eleonora × 2 dS m^−1^	46.72 ± 0.28	−9.99 ± 0.51	26.46 ± 0.78	30.46 ± 0.35 c	0.81 ± 0.00
Eleonora × 3 dS m^−1^	46.47 ± 0.23	−9.07 ± 0.22	22.95 ± 1.40	32.34 ± 0.50 ab	0.82 ± 0.00
Aroma 2 × 1 dS m^−1^	44.89 ± 0.28	−8.27 ± 0.14	19.58 ± 0.62	30.94 ± 0.71 bc	0.81 ± 0.01
Aroma 2 × 2 dS m^−1^	44.61 ± 0.51	−6.28 ± 0.46	13.50 ± 1.58	32.94 ± 0.45 a	0.82 ± 0.00
Aroma 2 × 3 dS m^−1^	44.58 ± 0.13	−5.74 ± 0.08	11.75 ± 0.08	33.76 ± 0.19 a	0.82 ± 0.00
Italiano Classico × 1 dS m^−1^	45.93 ± 1.25	−11.53 ± 0.42	31.87 ± 1.57	29.65 ± 0.47 c	0.79 ± 0.01
Italiano Classico × 2 dS m^−1^	46.16 ± 0.89	−11.37 ± 0.29	30.55 ± 0.94	31.22 ± 0.80 bc	0.81 ± 0.00
Italiano Classico × 3 dS m^−1^	46.27 ± 0.37	−10.90 ± 0.07	27.28 ± 0.32	31.02 ± 0.63 bc	0.80 ± 0.00
Significance					
Cultivar (C)	**	***	***	***	*
Nutrient solution (NS)	ns	***	***	***	*
C × NS	ns	ns	ns	*	ns

ns, *, **, *** Nonsignificant or significant at *p* ≤ 0.05, 0.01 and 0.001, respectively. Different letters within each column indicate significant differences according to Duncan’s multiple-range test (*p* = 0.05). All data are expressed as mean ± se (n = 3).

**Table 3:** Sweet basil aromatic profile in light of the cultivar and the nutrient solution electrical conductivity.

Source of Variance	Trans-2-Hexenal	1-Octen-3-ol	Eucalyptol	β-cis-Ocimene	Linalool	Eugenol	α-Bergamotene
	(%)	(%)	(%)	(%)	(%)	(%)	(%)
Cultivar (C)							
Eleonora	1.79 ± 0.18 b	4.44 ± 0.18 a	33.56 ± 1.42 b	1.99 ± 0.18 b	35.57 ± 1.73 a	5.78 ± 0.65 b	3.93 ± 0.48 a
Aroma 2	2.68 ± 0.16 a	3.08 ± 0.14 b	32.09 ± 1.26 b	2.45 ± 0.13 a	37.99 ± 1.54 a	3.59 ± 0.28 c	2.21 ± 0.22 b
Italiano Classico	2.82 ± 0.17 a	3.49 ± 0.10 b	45.13 ± 1.24 a	1.50 ± 0.14 c	20.74 ± 2.45 b	9.70 ± 1.33 a	3.61 ± 0.16 a
Nutrient solution concentration (NS)							
1 dS m^-1^	2.52 ± 0.12	3.65 ± 0.19	36.21 ± 2.07	1.80 ± 0.16 b	32.58 ± 3.51	7.42 ± 1.85 a	2.81 ± 0.36 b
2 dS m^-1^	2.39 ± 0.21	3.61 ± 0.26	38.34 ± 2.76	1.77 ± 0.21 b	30.05 ± 3.48	6.28 ± 0.76 ab	3.06 ± 0.34 b
3 dS m^-1^	2.38 ± 0.32	3.75 ± 0.28	36.24 ± 2.37	2.36 ± 0.17 a	31.67 ± 2.89	5.37 ± 0.62 b	3.89 ± 0.43 a
C × NS							
Eleonora × 1 dS m^−1^	2.34 ± 0.18 bc	4.34 ± 0.20	32.66 ± 1.70	1.76 ± 0.26	37.08 ± 2.01	4.49 ± 0.63 cde	2.86 ± 0.13 bc
Eleonora × 2 dS m^−1^	1.64 ± 0.23 cd	4.41 ± 0.39	33.50 ± 3.00	1.79 ± 0.37	35.05 ± 4.47	7.02 ± 1.07 bc	3.41 ± 0.79 b
Eleonora × 3 dS m^−1^	1.37 ± 0.24 d	4.56 ± 0.43	34.54 ± 3.40	2.41 ± 0.24	34.56 ± 3.18	5.84 ± 1.37 bcde	5.52 ± 0.35 a
Aroma 2 × 1 dS m^−1^	2.77 ± 0.26 ab	3.30 ± 0.07	32.68 ± 1.93	2.26 ± 0.20	40.04 ± 1.06	3.19 ± 0.53 e	1.59 ± 0.28 d
Aroma 2 × 2 dS m^−1^	2.86 ± 0.11 ab	2.85 ± 0.16	33.33 ± 2.49	2.35 ± 0.07	35.60 ± 2.49	3.96 ± 0.60 de	2.16 ± 0.11 cd
Aroma 2 × 3 dS m^−1^	2.41 ± 0.41 b	3.10 ± 0.40	30.27 ± 2.54	2.73 ± 0.30	38.34 ± 4.04	3.62 ± 0.36 e	2.88 ± 0.28 bc
Italiano Classico × 1 dS m^−1^	2.46 ± 0.17 b	3.29 ± 0.12	43.28 ± 2.69	1.39 ± 0.06	20.62 ± 5.73	14.58 ± 1.26 a	3.97 ± 0.15 b
Italiano Classico × 2 dS m^−1^	2.65 ± 0.23 ab	3.57 ± 0.12	48.19 ± 1.78	1.17 ± 0.16	19.48 ± 5.95	7.86 ± 1.12 b	3.60 ± 0.41 b
Italiano Classico × 3 dS m^−1^	3.35 ± 0.21 a	3.60 ± 0.25	43.91 ± 0.96	1.93 ± 0.21	22.12 ± 1.41	6.65 ± 0.43 bcd	3.26 ± 0.08 bc
Significance							
Cultivar (C)	***	***	***	***	***	***	***
Nutrient solution (NS)	ns	ns	ns	**	ns	*	**
C × NS	*	ns	ns	ns	ns	***	**

ns, *, **, *** Nonsignificant or significant at *p* ≤ 0.05, 0.01 and 0.001, respectively. Different letters within each column indicate significant differences according to Duncan’s multiple-range test (*p* = 0.05). All data are expressed as mean ± se (n = 3).

**Table 4:** Sweet basil phenolic acids profile in light of the cultivar and the nutrient solution electrical conductivity.

Source of Variance	Caffeic Acid	Chicoric Acid	Rosmarinic Acid	Ferulic Acid	Total Polyphenols
	(µg g^−1^ dw)	(µg g^−1^ dw)	(µg g^−1^ dw)	(µg g^−1^ dw)	(µg g^−1^ dw)
Cultivar (C)					
Eleonora	16.10 ± 1.04 c	32.74 ± 0.64 c	14.71 ± 1.61 c	2.96 ± 0.20 c	66.50 ± 1.65 c
Aroma 2	23.57 ± 1.58 b	50.54 ± 6.99 b	151.62 ± 18.01 b	12.07 ± 2.03 a	237.76 ± 15.41 b
Italiano Classico	50.67 ± 1.43 a	205.20 ± 31.70 a	326.03 ± 54.21 a	5.67 ± 0.55 b	587.49 ± 79.71 a
Nutrient solution concentration (NS)					
1 dS m^−1^	32.32 ± 6.14 a	121.30 ± 43.50 a	148.92 ± 45.81 b	7.67 ± 1.44 a	310.19 ± 93.99 b
2 dS m^−1^	29.68 ± 5.04 b	115.90 ± 31.01 a	218.21 ± 75.41 a	9.09 ± 2.42 a	372.81 ± 110.81 a
3 dS m^−1^	28.34 ± 4.92 b	51.30 ± 8.18 b	125.21 ± 31.21 b	3.94 ± 0.47 b	208.76 ± 37.33 c
C × NS					
Eleonora x 1 dS m^−1^	12.49 ± 0.24 f	33.35 ± 0.98 d	20.09 ± 0.83 e	3.40 ± 0.18 de	69.33 ± 0.03 e
Eleonora × 2 dS m^−1^	19.28 ± 1.07 de	32.88 ± 1.84 d	14.60 ± 1.36 e	3.10 ± 0.36 de	69.86 ± 1.85 e
Eleonora × 3 dS m^−1^	16.52 ± 0.43 e	31.98 ± 0.22 d	9.44 ± 0.35 e	2.37 ± 0.22 e	60.31 ± 0.63 e
Aroma 2 × 1 dS m^−1^	29.72 ± 0.65 c	35.25 ± 0.70 d	105.8 ± 2.77 d	12.68 ± 1.39 b	183.46 ± 5.33 d
Aroma 2 × 2 dS m^−1^	20.29 ± 0.83 de	78.17 ± 3.23 c	127.72 ± 10.21 d	18.29 ± 2.02 a	244.49 ± 12.67 c
Aroma 2 × 3 dS m^−1^	20.68 ± 0.46 d	38.19 ± 0.75 d	221.23 ± 5.43 c	5.23 ± 0.73 cde	285.34 ± 5.71 c
Italiano Classico × 1 dS m^−1^	54.74 ± 1.08 a	295.2 ± 7.31 a	320.93 ± 34.02 b	6.92 ± 1.02 c	677.78 ± 37.34 b
Italiano Classico × 2 dS m^−1^	49.48 ± 3.01 b	236.5 ± 7.82 b	512.23 ± 11.12 a	5.88 ± 0.87 cd	804.08 ± 8.68 a
Italiano Classico × 3 dS m^−1^	47.80 ± 1.02 b	83.74 ± 1.62 c	144.84 ± 12.83 d	4.23 ± 0.06 cde	280.62 ± 12.34 c
Significance					
Cultivar (C)	***	***	***	***	***
Nutrient solution (NS)	**	***	***	***	***
C × NS	***	***	***	***	***

**, *** Significant at *p* ≤ 0.01 and 0.001, respectively. Different letters within each column indicate significant differences according to Duncan’s multiple-range test (*p* = 0.05). All data are expressed as mean ± se (n = 3).
